# Editorial: Present and future of EMDR in clinical psychology and psychotherapy, volume II

**DOI:** 10.3389/fpsyg.2023.1138153

**Published:** 2023-02-24

**Authors:** Antonio Onofri

**Affiliations:** Centro Clinico de Sanctis, Roma, Italy

**Keywords:** EMDR therapy, EMDR research, eye movement desensitization and reprocessing, psychological trauma, PTSD, pandemic, COVID, Coronavirus

After the extraordinary success of the previous Frontiers Research Topic dedicated to “*Present and future of EMDR*” (~500,000 views), this is a sequel to the same topic, and is a result of increasing interest in this pioneering psychotherapeutic approach.

In total, 16 articles have been published and five co-editors, 77 authors, and a very large number of reviewers actively collaborated and contributed to this Research Topic on an almost daily basis.

In particular, during the COVID-19 pandemic (a period characterized by a large number of people exposed to an ongoing trauma), many studies were carried out, which demonstrated the feasibility of EMDR therapy applied online (i.e., Perri et al., 2021).

This enormously contributed to EMDR gaining popularity among professionals and interested people alike, becoming more known among the scientific community.

Hence, in this special second Research Topic, readers can find a large number of innovative articles (Lazzaroni, Invernizzi et al.; Mischler et al.; Farrell et al.; Yurtsever et al.; Fernandez et al.; Faretta et al.; Lazzaroni, Tossi et al.) that document how EMDR therapy, applied during the Coronavirus period, helped to relieve stress in healthcare workers, patients, and their family members.

Immediately after its birth in the last decade of the last century, several meta-analyses investigated real evidence for EMDR therapy in treating post-traumatic stress disorder (PTSD) (Jonas et al., 2013; Watts et al., 2013; Chen et al., 2014, 2015; Wilson et al., 2018; de Jongh et al., 2019; Yunitri et al., 2020; Carletto et al., 2021).

Based on the outcomes of these investigations, EMDR was included in many international clinical guidelines and was even recognized by the World Health Organization in 2013 as well as finally being aknowledged as an elective treatment by the U. S. Veterans Affairs Department (2017).

In this special Frontiers Research Topic, scholars will find and appreciate several interesting articles dedicated to the field of trauma and stress-related conditions; from child-birth to traffic accidents and domestic violence (Brouwers et al.; Kranenburg et al.; Burak Yaşar et al.; Susanty et al.; Rodriguez-Garay and Mosquera).

It is of crucial importance to mention that, for the two last decades, the application of EMDR is no longer strictly limited to the treatment of PTSD: its feasibility has been expanded to the treatment of other psychiatric disorders and conditions (de Bont et al., 2013; Novo et al., 2014; Perez-Dandieu and Tapia, 2014).

In fact, the relevance of the so-called “post-traumatic psychopathological dimension” and the clinical importance of traumatic events or traumatic prolonged conditions, have been considered as crucial risk factors for the development of many different somatic and psychiatric disorders, especially if these occurred during infancy and childhood (Kim and Lee, 2016; Millan et al., 2017).

Based on this rather new awareness (the connection between somatic conditions and post trauma disorders), clinicians and researchers are revising their diagnoses of PTSD, allowing them to better address the seriousness of any somatic or psychiatric condition in a safer manner (Assion et al., 2009). This is probably one of the main reasons that persuaded many of them, all over the world, to consider EMDR as a useful therapy not only for PTSD but also for several other pscychological or psychiatric conditions.

Thus, the application of EMDR has been continuously growing, and, as a consequence, the need for more randomized controlled trials with it.

Therefore, many studies have recently investigated the effect of EMDR on other mental health conditions such as domestic violence, traumatic grief, traumatic cancer diagnosis, emergency situations, psychotic disorder, depressive and bipolar disorders, anxiety, addiction, and chronic pain.

The data available from studies on some of these disorders, for example, phobias (De Jongh and ten Broeke, 2007), present a consistent and relevant outcome, making it possible that, in future, EMDR will also be considered as an evidence-based therapy for these conditions.

Moreover, evidence has shown EMDR to be a promising effective therapy for depressive disorders (Hase et al., 2015; Valiente-Gomez et al., 2017).

As proof of this, readers can come across an interesting article in this Research Topic by Altmeyer et al. presenting how EMDR therapy leads to a high rate of remission and a decrease in the number of relapses in patients with depressive disorders.

Interestingly, EMDR also seems to constitute a helpful tool to promote psychological and somatic improvement in patients with organic diseases, for example, suffering from depressive symptoms as a consequence of heart attack (Behnammoghadam et al., 2015).

Regarding disorders other than PTSD, EMDR has also shown positive effects in improving OCD symptoms (Feske and Goldsteina, 1997; Nazari et al., 2011; Doering et al., 2013; Triscari et al., 2015). In terms of addictive disorders, alcohol dependence, and craving, EMDR has been able to facilitate a good outcome in this clinical population (Hase et al., 2008; Perez-Dandieu and Tapia, 2014).

It has also been considered a safe and effective therapeutic treatment in decreasing the intensity of conditions such as chronic back pain, which can affect a good portion of society at some stage in their lives and is always relevant.

Last but not least, the application of EMDR in psychotic disorders constitutes the opening of a new frontier in clinical research (Valiente-Gomez et al., 2017).

In summary, EMDR therapy can be seen as an useful tool and an appropriate therapy for various psychiatric conditions associated with PTSD, and as a safe treatment, with no relevant side effects (Feske and Goldsteina, 1997; Doering et al., 2013; Novo et al., 2014; Perez-Dandieu and Tapia, 2014; Hase et al., 2015; Triscari et al., 2015; van den Berg et al., 2015; Gerhardt et al., 2016).

Beyond the clinical point of view, interest in understanding how EMDR actually works (the neurophysiological pathways behind it) has greatly increased in recent years.

As highlighted by Castelnuovo et al. (2019) in their Editorial in the previous Research Topic on this field (*Present and future of EMDR*), two recent articles have gone a step further and are highly relevant to the field.

One, published in *Nature* by Baek et al. (2019), reveals EMDR's mechanism of action and neuroanatomical pathway using an animal model. The authors found that bilateral stimulation, as compared to controlled conditions, led to a clear and persistent decrease in fear behavior. Furthermore, the authors observed that bilateral stimulation increased neuronal activity in the superior colliculus and the mediodorsal thalamus, thus dampening the excitability of neurons in the basolateral nucleus of the amygdala.

The other article is a review published in *Neuron* by Maddox et al. (2019) on the encoding of aversive memory.

Also in this Research Topic, readers will find a very engaging paper on the neurophysiological mechanisms implicated in EMDR (Mattera et al.): it deeply investigates the PTSD mechanisms and hypothesizes how EMDR achieves trauma relief. The authors used a biologically inspired computational model, based on firing rate units, comparing data from patients treated with EMDR or prolonged exposure.

Finally, I would like discuss the innovative article by Hase and Brisch on EMDR and its therapeutic relationship. In this article, the two authors try to describe the therapeutic relationship in EMDR therapy from an attachment-based perspective. The authors conclude their article by describing EMDR therapy as a sensitive psychotherapy, discussing the implications for the treatment, training, and research.

Therefore, it is my absolute pleasure to present this second special Research Topic in *Frontiers in Psychology*, completely dedicated to EMDR.

## Author contributions

The author confirms being the sole contributor of this work and has approved it for publication.

## References

[B1] Assion H. J. Brune N. Schmidt N. Aubel T. Edel M. A. Basilowski M.. (2009). Trauma exposure and post-traumatic stress disorder in bipolar disorder. Soc. Psychiatry Psychiatr. Epidemiol. 44, 1041–1049. 10.1007/s00127-009-0029-1 19434346

[B2] Baek J. Lee S. Cho T. Kim S. W. Kim M. Yoon Y.. (2019). Neural circuits underlying a psychotherapeutic regimen for fear disorders. Nature 566, 339–343. 10.1038/s41586-019-0931-y 30760920

[B3] Behnammoghadam M. Alamdari A. K. Behnammoghadam A. Darban F. (2015). Effect of eye movement desensitization and reprocessing (EMDR) on depression in patients with myocardial infarction (MI). Glob. J. Health Sci. 7, 258–262. 10.5539/gjhs.v7n6p258 26153191 PMC4803841

[B4] Carletto S. Malandrone F. Berchialla B. Oliva F. Colombi N. Hase M.. (2021). Eye movement desensitization and reprocessing for depression: a systematic review and meta-analysis Eur. J. Psychotraumatol. 9, 1894736. 10.1080/20008198.2021.1894736 33889310 PMC8043524

[B5] Castelnuovo G. Fernandez I. Amman B. (2019). Editorial: present and future of EMDR in clinical psychology and psychotherapy. Front. Psychol. 10:2185. 10.3389/fpsyg.2019.02185 31611832 PMC6776929

[B6] Chen Y. R. Hung K. W. Tsai J. C. Chu H. Chung M. H. Chen S. R.. (2014). Efficacy of eye movement desensitization and reprocessing for patients with post-traumatic stress disorder: a meta-analysis of randomized controlled trials. PLoS ONE 9, e103676. 10.1371/journal.pone.0103676 25101684 PMC4125321

[B7] Chen Y. R. Zhang G. Hu M. Liang X. (2015). Eye movement desensitization and reprocessing vs. cognitive-behavioral therapy for adult post-traumatic stress disorder: systematic review and meta-analysis. J. Nerv. Mental Disord. 203, 443–451. 10.1097/NMD.0000000000000306 25974059

[B8] de Bont P. A. van Minnen A. de Jongh A. (2013). Treating PTSD in patients with psychosis: a within- group controlled feasibility study examining the efficacy and safety of evidence-based PE and EMDR protocols. Behav. Ther. 44, 717–730. 10.1016/j.beth.2013.07.002 24094795

[B9] De Jongh A. ten Broeke E. (2007). Treatment of specific phobias with EMDR: conceptualization and strategies for the selection of appropriate memories. J. EMDR Pract. Res. 1, 46–56. 10.1891/1933-3196.1.1.46

[B10] de Jongh A.d. Amann B. Hofmann A. Farrell D. Lee C. W. (2019). The status of EMDR therapy in the treatment of posttraumatic stress disorder 30 years after its introduction. J. EMDR Pract. Res. 13, 4. 10.1891/1933-3196.13.4.261

[B11] Doering S. Ohlmeier M. C. de Jongh A. Hofmann A. Bisping V. (2013). Efficacy of a trauma-focused treatment approach for dental phobia: a randomized clinical trial. Eur. J. Oral Sci. 121, 584–593. 10.1111/eos.12090 24206075

[B12] Feske U. Goldsteina J. (1997). Eye movement desensitization and reprocessing treatment for panic disorder: a controlled outcome and partial dismantling study. J. Consult. Clin. Psychol. 65, 1026–1035. 10.1037/0022-006X.65.6.1026 9420364

[B13] Gerhardt A. Leisner S. Hartmann M. Janke S. Seidler G. H. Eich W.. (2016). Eye movement desensitization and reprocessing vs. treatment-as-usual for non-specific chronic back pain patients with psychological trauma: a randomized controlled pilot study. Front. Psychiatry 7, 201. 10.3389/fpsyt.2016.00201 28066274 PMC5167699

[B14] Hase M. Balmaceda U. M. Hase A. Lehnung M. Tumani V. Huchzermeier C.. (2015). Eye movement desensitization and reprocessing (EMDR) therapy in the treatment of depression: a matched pairs study in an inpatient setting. Brain Behav. 5, 6. 10.1002/brb3.342 26085967 PMC4467776

[B15] Hase M. Schallmeyer S. Sacxk M. (2008). EMDR reprocessing of the addiction memory: pretreatment, posttreatment, and 1-month follow-up. J. EMDR Pract. Res. 2, 170–179. 10.1891/1933-3196.2.3.170

[B16] Jonas D. E. Cusack K. Forneris C. A. Wilkins T. M. Sonis J. Middleton J. C.. (2013). Psychological and Pharmacological Treatments for Adults With Posttraumatic Stress Disorder (PTSD). Rockville, MD: Agency for Healthcare Research and Quality (US) (2013). 23658937

[B17] Kim J. Lee S. H. (2016). Influence of interactions between genes and childhood trauma on refractoriness in psychiatric disorders. Progr. NeuroPsychopharmacol. Biol. Psychiatry 70, 162–169. 10.1016/j.pnpbp.2016.01.013 26827636

[B18] Maddox S. A. Hartmann J. Ross R. A. Ressler K. J. (2019). Deconstructing the gestalt: mechanisms of fear, threat, and trauma memory encoding. Neuron 102, 60–74. 10.1016/j.neuron.2019.03.017 30946827 PMC6450587

[B19] Millan M. J. Ricca V. Oliver D. Kingdon J. Valmaggia I. McGuire P. (2017). Deconstructing vulnerability for psychosis: metaanalysis of enviromental risk factors for psychosis in subjects at ultra-high risk. Eur. Pychiatry 40, 65–75. 10.1016/j.eurpsy.2016.09.003 27992836

[B20] Nazari H. Momeni N. Jariani M. Tarrahi M. J. (2011). Comparison of eye movement desensitization and reprocessing with citalopram in treatment of obsessive-compulsive disorder. Int. J. Psychiatry Clin. Pract. 15, 270–274. 10.3109/13651501.2011.590210 22122001

[B21] Novo P. Landin-Romero R. Radua J. Vicens V. Fernandez I. Garcia F.. (2014). Eye movement desensitization and reprocessing therapy in subsyndromal bipolar patients with a history of traumatic events: a randomized, controlled pilot-study. Psychiatry Res. 30, 122–128. 10.1016/j.psychres.2014.05.012 24880581

[B22] Perez-Dandieu B. Tapia G. (2014). Treating trauma in addiction with EMDR: a pilot study. J. Psychoact. Drugs 46, 303–309. 10.1080/02791072.2014.921744 25188700

[B23] Perri R. L. Castelli P. La Rosa C. Zucchi T. Onofri A. (2021). COVID-19, Isolation, quarantine: on the efficacy of internet-based eye movement desensitization and reprocessing (EMDR) and cognitive-behavioral therapy (CBT) for ongoing trauma. Brain Sci. 11, 579. 10.3390/brainsci11050579 33946179 PMC8145551

[B24] Triscari M. T. Faraci P. Catalisano D. D'Angelo V. Urso V. (2015). Effectiveness of cognitive behavioral therapy integrated with systematic desensitization, cognitive behavioral therapy combined with eye movement desensitization and reprocessing therapy, and cognitive behavioral therapy combined with virtual reality expo. Neuropsychiatr. Dis. Treat. 11, 2591–2598. 10.2147/NDT.S93401 26504391 PMC4605250

[B25] U. S. Veterans Affairs Department (2017). Management of Posttraumatic Stress Disorder and Acute Stress Reaction. Washington, DC: VA/DoD Clinical Practice Guidelines, Mental Health Guidelines.

[B26] Valiente-Gomez A. Moreno-Alcazar A. Treen D. Cedron C. Colom F. Perez V.. (2017). EMDR beyond PTSD: a systematic literature review. Front. Psychol. 8:1668. 10.3389/fpsyg.2017.01668 29018388 PMC5623122

[B27] van den Berg D. P. de Bont P. A. van der Vleugel B. M. de Roos C. de Jongh A. Van Minnen A.. (2015). Prolonged exposure vs eye movement desensitization and reprocessing vs waiting list for posttraumatic stress disorder in patients with a psychotic disorder: a randomized clinical trial. JAMA Psychiatry 72, 3. 10.1001/jamapsychiatry.2014.2637 25607833

[B28] Watts B. V. Schnurr P. P. Mayo L. Young-Xu Y. Weeks W. B. Friedman M. J. J. (2013). Meta-analysis of the efficacy of treatments for posttraumatic stress disorder. Clin. Psychiatry 74, 541–550. 10.4088/JCP.12r08225 23842024

[B29] Wilson G. Farrell D. Barron I. Hutchins J. Whybrow D. Kiernan M. D. (2018). The use of eye-movement desensitization reprocessing (EMDR) therapy in treating post-traumatic stress disorder-a systematic narrative review. Frontiers 6, 923. 10.3389/fpsyg.2018.00923 29928250 PMC5997931

[B30] Yunitri N. Kao C.-C. Chu H. Voss J. Chiu H.-L. Liu D.. (2020). The effectiveness of eye movement desensitization and reprocessing toward anxiety disorder: a meta-analysis of randomized controlled trials. J. Psychiatr. Res. 123, 102–113. 10.1016/j.jpsychires.2020.01.005 32058073

